# Accuracy of point-of-care coagulation testing during cardiopulmonary bypass in a patient post COVID-19 infection

**DOI:** 10.1186/s13019-022-01862-2

**Published:** 2022-05-07

**Authors:** Nimrat Grewal, David Yousef, Meindert Palmen, Robert Klautz, Jeroen Eikenboom, Jeroen Wink

**Affiliations:** 1 Department of Cardiothoracic Surgery, Leiden University Medical Center (LUMC), Albinusdreef 2, 2333 ZA Leiden, The Netherlands; 2 Division of Thrombosis and Hemostasis, Department of Internal Medicine, Leiden University Medical Center (LUMC), Leiden, The Netherlands; 3 Department of Anesthesiology, Leiden University Medical Center (LUMC), Leiden, The Netherlands

**Keywords:** Cardiopulmonary bypass, Coagulation testing, COVID-19, Case report

## Abstract

**Introduction:**

Extracorporeal circulation (ECC) in cardiac surgery is performed under systemic heparinization. Adequacy of heparin therapy and anticoagulation during ECC is assessed by activated clotting time (ACT), although there are concerns regarding the reliability of this measure. The ACT can be affected by factors other than heparin anticoagulation. A novel factor that should be considered is the influence of a COVID-19 infection. More than half of the hospitalized COVID-19 patients develop coagulation abnormalities with dysregulated coagulation test results. Patients recently recovered from COVID-19 may still demonstrate some forms of coagulation disorder affecting the ACT. This case describes an inaccurate point-of-care ACT testing in a patient with previous COVID-19 infection undergoing cardiac surgery with ECC and the alternative coagulation testing performed.

**Case presentation:**

A 77-years-old Caucasian male presented with symptomatic severe mitral valve regurgitation for which he underwent surgery. Medical history revealed a COVID-19 infection one month before surgery. Pre-operative hematological lab results were normal and baseline ACT during surgery was 100 s. To achieve an adequate ACT of > 400 s, multiple doses of heparin were needed and after administration of a triple dose (75,000 IE heparin in total) this adequate ACT was achieved. In the meanwhile we measured anti-Xa level and APTT, which were at adequate levels when ACT was still < 400 s.

**Discussion:**

This case emphasizes the need of alternative methods for monitoring heparin therapy in case ACT does not respond adequately. Another point to highlight in this case is the poorly correlated relation between ACT and APTT and anti-Xa in light of the recent COVID-19 infection. Although studies have shown that COVID-19 infection can cause coagulopathy and altered hemostatic parameters, ACT has never been investigated in COVID-19 patient. Understanding the correlation between ACT, APTT and anti-Xa in COVID-19 patients is mandatory.

## Introduction

Cardiac surgery, which requires extracorporeal circulation, is performed under systemic heparinization. Adequate anticoagulation and its reversal by protamine is essential for the safe conduct of surgical procedures requiring cardiopulmonary bypass. Inadequate anticoagulation can lead to serious untoward events, including oxygenator blockage causing cessation of extracorporeal support and multiorgan failure due to micro-emboli [1]. On the other hand, excessive heparinization results in increased postoperative bleeding [2, 3]. Heparin anticoagulation during cardiopulmonary bypass may be monitored by measuring heparin effect. Earlier, whole blood clotting time was used to monitor the anticoagulant effect of heparin and ever since the activated partial thromboplastin time (APTT) and anti-factor-Xa assays are considered the gold standard for monitoring heparin induced anticoagulation [4]. However, in cardiac surgery these assays are not useful intraoperatively due to long turnaround times. The standard method to assess the adequacy of heparin therapy and anticoagulation during extracorporeal circulation, be this extracorporeal life support or cardiopulmonary bypass, is therefore measuring the heparin effect by using the activated clotting time (ACT). The ACT is a point of care whole blood assay that measures the time in seconds taken for a blood sample to clot after it has been exposed to a catalyst activating the intrinsic pathway [1, 5]. The ACT test has existed for over 5 decades [6] and has been used in the context of cardiac surgery since the 1970s [7–9]. Even though the ACT is considered the clinical standard for intraoperative point of care monitoring of heparin anticoagulation during cardiac surgery, there are concerns regarding the reliability of this measure. Firstly, the ACT is affected by other factors during cardiopulmonary bypass that are not associated with heparin anticoagulation, including hemodilution, hypothermia and thrombocytopenia [10]. Secondly, concerns exist regarding reproducibility of ACT assays and comparability of ACT measurement devices [11]. Another factor which could influence the ACT and should be considered in this time is the influence of the coronavirus disease 2019 (COVID-19) on the coagulation tests. While the majority of critically ill patients with COVID-19 have isolated respiratory failure, around 60–70% of the hospitalized patients develop coagulation abnormalities, such as thrombocytopenia, hypercoagulation, disseminated intravascular coagulation (DIC) and venous thrombosis, with corresponding dysregulated coagulation tests including high fibrinogen levels leading to short APTTs [12, 13]. With the increasing number of COVID-19 infections worldwide, chances are high that patients requiring cardiac surgery have recently recovered from a coronavirus infection and still demonstrate some forms of coagulation disorders. Many studies are currently focusing on possible pathophysiological mechanisms leading to coagulopathy during active COVID-19 infection [14]. Less is however known about the long-term consequences of the COVID-19 related coagulopathy.

In this paper we present a case of inaccurate point-of-care ACT testing in a patient with previous COVID-19 infection undergoing cardiac surgery requiring extracorporeal circulation and describe what alternative coagulation testing was performed.

## Case presentation

### Admission and preoperative work-up

A 77-year-old Caucasian male presented with chest discomfort and progressive exertional dyspnea, for which he was admitted to the hospital. Medical history of the patient revealed hypertension, hypercholesterolemia, acute coronary syndrome for which a percutaneous coronary intervention of the left descending artery was performed (2016) and atrial flutter, converted to sinus rhythm after electrocardioversion. He was SARS-CoV-2 PCR positive on nasopharyngeal swab a month before surgery, with minor symptoms of cold. He developed pulmonary embolism, for which oral anticoagulation (dabigatran) was initiated. His surgical, family and social histories were unremarkable.

Upon admission, physical examination revealed a systolic murmur, most pronounced at the apex, in a clinically stable patient with adequate vital functions. The chest was clear with equal air entry and no added sounds. No other physical abnormalities were noted.

ECG showed sinus rhythm, with normal conduction times and no signs of ischemia. Echocardiography (transthoracic and transesophageal) revealed severe mitral valve regurgitation caused by a prolapse of the P2 segment of the posterior leaflet with flail leaflet. Chordal rupture was also noted. In addition, moderate tricuspid regurgitation and a tricuspid annular diameter of 38 mm was observed. The left ventricle was non-dilated and hypertrophied, with good systolic function. Coronary angiography showed a significant stenosis of the left circumflex artery. In corroboration with interventional cardiologist and imaging cardiologist, the patient was accepted for mitral valve repair, tricuspid valve repair and a single coronary artery bypass graft on the left circumflex artery. The anticoagulant dabigatran was discontinued 5 days prior to surgery. Preoperative complete blood count showed a platelet count of 192 × 10^9^/L (normal range, 150–400 × 10^9^/L), activated partial thromboplastin time (APTT) of 31.1 s (normal range 24.7–31.7 s), prothrombin time (PT) 13.9 s (normal range 12.5–14.9 s) and an INR of 1.0.

### Surgical procedure

The baseline ACT value in our patient was 100 s. After sternotomy the surgeon requested systemic heparinization to be administered to achieve an ACT > 400 s, the cut off for adequate heparinization required for extracorporeal circulation in our practice. After 25,000 IU (312 IU/kg) of heparin was given the ACT was 363 s. Additional doses of 5000 IU and subsequently 10,000 IU of heparin were administered but failed to increase ACT values. To rule out measurement error we used three separate devices [all Hemochron Signature Elite (Instrumentation Laboratory, Bedford, MA 01730-2433 USA)]. During multiple ACT measurements all devices demonstrated ACT values < 400 s, except for one measurement with one of the three devices that showed an ACT of > 600 s. We suspected that the ACT might not be in an adequate range after extra boluses heparin due to an antithrombin deficiency. Therefore, as per our protocol, we decided to administer two units of fresh frozen plasma after which the ACT still did not increase. We gave an extra bolus of heparin (10,000 IU) and two units of fresh frozen plasma. In the meanwhile we consulted the hematologist. As in COVID-19 patients dysregulation of the ACT/APTT is described, we were advised to measure the anti-Xa level and APTT in plasma. While awaiting the laboratory results, ACT was again measured, which was still < 400 s. We administered an additional bolus of 25,000 IE of heparin. After an hour the lab values were available: APTT > 120 s, heparin level in serum > 2.00 IU/mL, antithrombin activity 88% (normal range 84–116%). The ACT level was now also > 500 s. In turn, we could then start the cardiopulmonary bypass and perform the surgery.

### Postoperative period

After admission to the intensive care unit patient experienced excessive blood loss (1400 cc) which was not responsive to administration of Fresh Frozen Plasma (2 units), blood platelets (1 unit) and tranexamic acid. Rethoracotomy was performed, however a bleeding focus was not found. After the re-operation an additional 1100 cc of blood loss was observed. Patient went to the ward on the first postoperative day. Two units of packed red cells were administered in the early postoperative period because of low hemoglobin levels. Further hospital stay went uneventful.

## Discussion

In cardiac surgery measuring the ACT is considered the standard method to assess the adequacy of heparin therapy and anticoagulation during extracorporeal circulation, be this extracorporeal life support and cardiopulmonary bypass, or during interventional procedures in the catheter laboratory. The gold standard of assessing heparin concentration is the anti-Xa assay and APTT. These assays however require plasma instead of whole blood, and results are not immediately available to the clinician, making it nearly impossible to use these tests during cardiac surgery. There is enormous practice variability relating to the use and dosing of heparin, monitoring heparin anticoagulation, reversal of anticoagulation and the use of alternative anticoagulants. In 2017 the Society of Thoracic Surgeons (STS), the Society of Cardiovascular Anesthesiologists (SCA), and the American Society of Extracorporeal Technology (AmSECT) developed evidence-based practice guidelines which described the optimal management of anticoagulation during the conduct of cardiopulmonary bypass (CPB) [15]. These guidelines define a threshold of > 480 s as an adequate ACT during CPB [15]. However, this minimum threshold value is an approximation and may vary based upon the bias of the instrument being used. For instruments using ‘maximal activation’ of whole blood or microcuvette technology, values above 400 s are frequently considered therapeutic (Class 2a recommendation, LOE C). The ACT has been regarded the clinical standard in cardiac surgery ever since it has been described by Hattersley [5], although the reliability of this test is questionable. Also, the reproducibility of ACT tests using simultaneous measurements on identical device types and comparison of results between different device types are of great concern. Dirkmann et al. recently compared the agreement of ACT assays using four parallel measurements performed on two commonly used devices each (i.e., two Hemochron Signature Elite (Hemochron) and two Abbott i-STAT (i-STAT) devices, respectively) [11]. This study concluded that currently used ACT point-of-care devices cannot be used interchangeably due to marked differences between both identical and different device types. The reliability of the Hemochron in assessing adequacy of heparin anticoagulation monitoring for CPB was further questioned [11].

Here we describe a case of heparin resistance, which is defined as the failure of unusually high doses of heparin to achieve a target ACT (i.e. > 400 s) [16]. In case of a clinical scenario of heparin resistance, described therapeutic approaches in the literature are administering additional heparin, supplement antithrombin with FFP, supplement antithrombin with an antithrombin concentrate or accept the ACT and proceed with CPB without any additional treatment [16]. Conventionally, as in our case additional heparin has been administered until the ACT reaches target values [16], although Levy et al. has shown in vitro that whole blood heparin concentrations > 4.1 units/mL fails to further increase the ACT [17].

We had to administer a three times higher dosage of heparin in order to achieve an adequate ACT. In our patient the ACT values did not correlate with the APTT and anti-Xa. Guidance on basis of ACT values, which were consistently inadequate, led to overdosing of heparin (624 IU/kg) in the patient, evidenced by an APTT of > 120 s and anti-Xa > 2 IU/mL. By the time the measured APTT and anti-Xa level were more than adequate, the ACT still lagged behind. Interestingly, in our case during the repeated ACT measurements with three devices [Hemochron Signature Elite (Instrumentation Laboratory, Bedford, MA 01730-2433 USA)], all indicated ACT values < 400 s, except for a single measurement which showed an ACT value of > 600 s. The key-point is that start of cardiopulmonary bypass was assumed safe when ACT value was > 400. However, in order to achieve an ACT > 400 we administered an overdose of heparin and consequently introduced an increased risk of postoperative bleeding [1, 2].

In the literature the described correlation between APTT, anti-Xa and ACT is also highly variable [18]. Smythe et al. found that decisions to adjust heparin therapy based on ACT results differed from decisions based on APTT results more than one-third of the time [18]. Faraoni et al. also concluded in their study that ACT and APTT correlated poorly during endovascular treatment of cerebral aneurysms [19]. This case emphasizes the need of alternative monitoring methods for heparin such as anti-Xa, when the ACT does not respond.

**Fig. 1:**
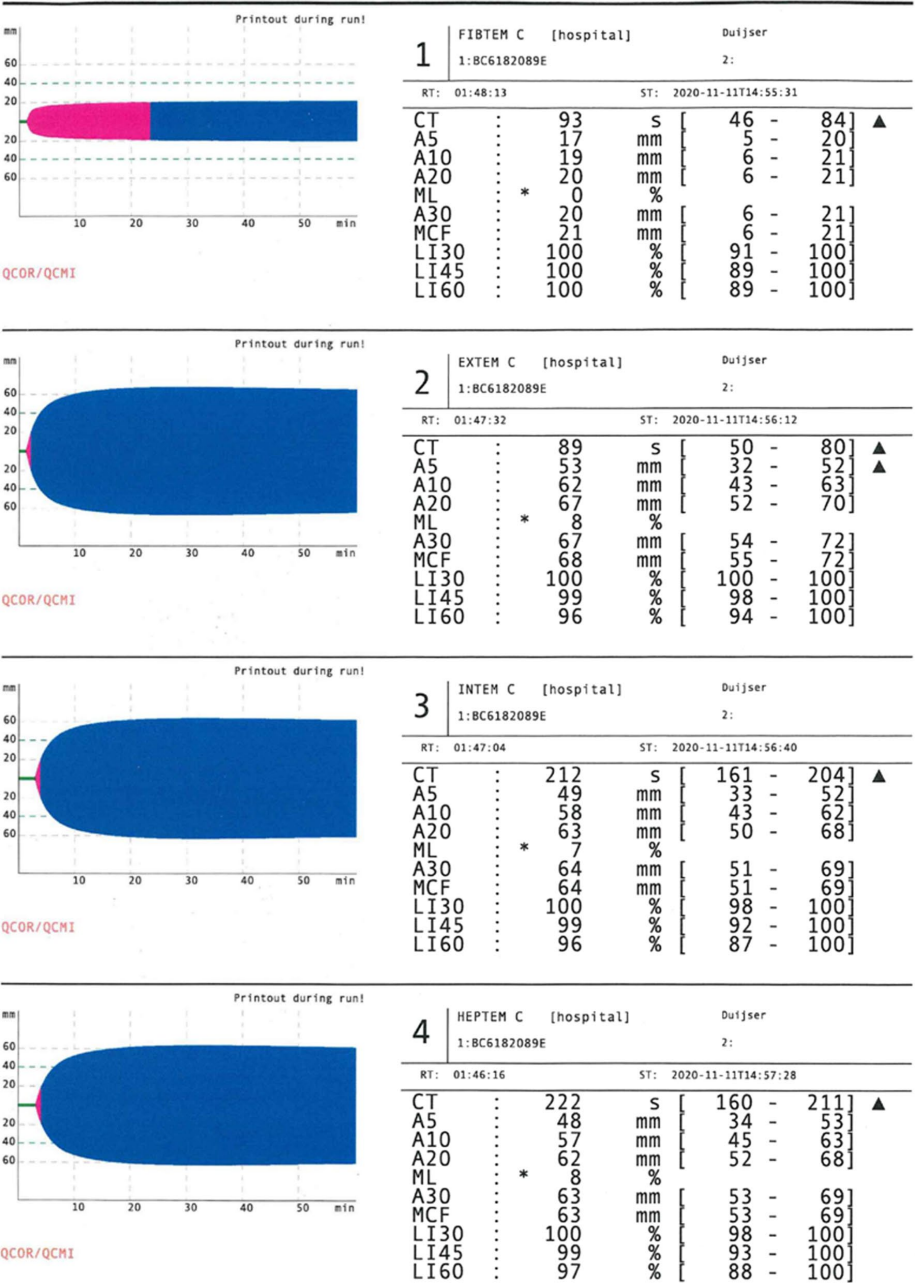
ROTEM analysis after protamine. In HEPTEM, coagulation is activated as in INTEM (via the contact phase). In HEPTEM, however, the addition of heparinase in the reagent degrades heparin present in the sample. The comparison of INTEM and HEPTEM allows for a specific detection of a heparin effect. The clotting time (CT) in HEPTEM as compared to INTEM is not shortened, indicating no residual heparin effect

A point of interest in this case is the required amount of protamine for the heparin reversal. Daily practice is to use a ratio of protamine to heparin of 1:1. We, however, chose to carefully titrate the protamine dose to limit the protamine-to-heparin ratio and started with administration of 300 mg (30.000 IU) protamine. This already resulted in adequate heparin reversal evidenced by an ACT value of 112 s. Adequate heparin reversal by protamine was confirmed by ROTEM analysis (ROTEM Sigma, Bedford, MA 01730-2433 USA) (Fig. 1). An APTT of 39.5 s and anti-Xa of 0.11 IU/mL indicated minimal residual heparin effects, which were accepted because of surgical hemostasis.

Another interesting angle in this case is the patients history of COVID-19. Careful study of the hemostatic parameters has revealed common disturbances of laboratory values in most COVID-19 positive patients. The PT, INR and thrombin time (TT) are commonly prolonged, whereas the APTT is variably described shortened and prolonged [14]. High fibrinogen and factor VIII levels, which are often encountered in COVID-19 patients, are associated with shorter APTT [13, 20]. In our patient the APTT and anti-Xa levels at the start of cardiopulmonary bypass were high in therapeutic range, likely ruling out residual effects COVID-19 on coagulation. The ACT has however not been studied in COVID-19 patients yet, therefore we do not know what effect can be expected on this laboratory value. In our case the ACT correlated extremely poor with the APTT and anti-Xa level. Despite adequate anticoagulation after 612 mg/kg heparin, ACT values measured by multiple devices failed to reach values > 400, reflecting severe false low values. As most studies have focused on coagulopathy in patients with active COVID-19 infection and ACT has not been investigated in this patient population, it is difficult to address the observed discrepancy between the ACT and APTT/anti-Xa. With the increasing number of COVID-19-infected patients, the share of previously infected patients undergoing cardiac surgery will only rise. As the ACT monitoring is our standard monitoring tool for patients on extracorporeal circulation, understanding the correlation between ACT, APTT and anti-Xa and the link with the biology in COVID-19 patients is of utmost importance. This warrants further study of the relationship between ACT, APTT and anti-Xa in COVID-19 patients.

### Take home message

In cardiac surgery measuring the ACT is considered the standard method to assess the adequacy of heparin therapy and anticoagulation during extracorporeal circulation, be this extracorporeal life support and cardiopulmonary bypass. The gold standard of assessing heparin concentration is the anti-Xa assay and APTT. This case emphasizes the need of alternative monitoring methods for heparin such as anti-Xa, when the ACT does not respond.

## Data Availability

Yes.
